# Socio-demographic, agricultural, and personal protective factors in relation to health literacy among farmers from all regions of Thailand

**DOI:** 10.3389/fpubh.2024.1364296

**Published:** 2024-03-25

**Authors:** Ratana Sapbamrer, Nalin Sittitoon, Sakesun Thongtip, Eakasit Chaipin, Chatchada Sutalangka, Aroon La-up, Phiman Thirarattanasunthon, Ajchamon Thammachai, Boonsita Suwannakul, Noppharath Sangkarit, Amornphat Kitro, Jinjuta Panumasvivat, Taweewun Srisookkum

**Affiliations:** ^1^Department of Community Medicine, Faculty of Medicine, Chiang Mai University, Chiang Mai, Thailand; ^2^Environmental and Occupational Medicine Excellence Center (EnOMEC), Faculty of Medicine, Chiang Mai University, Chiang Mai, Thailand; ^3^School of Environmental Health, Institute of Public Health, Suranaree University of Technology, Nakhon Ratchasima, Thailand; ^4^Department of Environmental Health, School of Public Health, University of Phayao, Phayao, Thailand; ^5^Department of Public Health, Faculty of Science, Rajabhat Lampang University, Lampang, Thailand; ^6^Department of Physical Therapy, School of Integrative Medicine, Mae Fah Luang University, Chiang Rai, Thailand; ^7^Nakhonsawan Campus, Mahidol University, Nakhonsawan, Thailand; ^8^School of Public Health, Walailak University, Nakhon Si Thammarat, Thailand; ^9^Department of Physical Therapy, School of Allied Health Sciences, University of Phayao, Phayao, Thailand; ^10^Department of Community Health, School of Public Health, University of Phayao, Phayao, Thailand

**Keywords:** health literacy, pesticide, personal protective behavior, farmer, agricultural worker

## Abstract

**Introduction:**

Farmers are vulnerable to adverse health effects from pesticide exposure due to their health literacy (HL). Therefore, this study aims to investigate HL among farmers in four main regions of Thailand, investigating socio-demographics, agricultural, and personal protective factors to their HL.

**Methods:**

This cross-sectional design study was conducted on 4,035 farmers from January to July 2023. The European Health Literacy Survey Questionnaire-47 items were used to measure HL.

**Results:**

Thai farmers had a mean HL score of 34.7 ± 8.7, and the farmers in the North region of Thailand had the highest frequency of limited HL (75.8%). Socio-demographic factors that were associated with HL included gender, region of living, marital status, education level, co-morbidity, and number of family members. Agricultural factors associated with HL included planting status, working hours on farm, spraying pesticides, harvesting crops, pesticide use of >1 type, access information from government officers, access information from posters/brochures, information from online multimedia, and access information from neighbors. Personal protective factors that were associated with HL included wearing a hat, goggles, a rubber apron, and a long-sleeved shirt.

**Discussion:**

Our study recommends that strategies and interventions to enhance the HL of farmers should be focused on the target populations, which include men, widows, or divorced, those with low levels of education, those who have co-morbidities, and those who applied pesticides of more than 1 type and improper personal protective equipment (PPE) use. The primary emphasis needs to be on the North region of Thailand, making that the target area to improve health equity in Thailand. These efforts would enhance the HL of farmers and sustainably improve pesticide safety behavior. Additionally, there is an urgent need for supportive measures aimed at altering on-farm practices and promoting education on alternative pest management strategies, particularly non-chemical crop protection, to ensure sustainable agriculture.

## 1 Introduction

Health literacy (HL) concerns the knowledge and competencies of individuals to obtain, understand, and use information and services necessary to make appropriate health-related decisions (1, 2). HL is crucial for empowering people, prioritizing health concerns, and implementing health promotion at local, national, and global levels (3, 4). The European HL Survey Questionnaire-47 items (HLS-EU-Q47) was developed to assess HL in a population based on a conceptual framework of three contexts of health domains (health care, disease prevention, and health promotion) across four information processing dimensions (access, understand, appraise, and apply) (5). It has been used and validated worldwide across Europe, Asia, and some other countries (6–9). Most previous studies investigated HL among the general population and older people, and also socio-demographic factors. However, there is a notable gap in the literature concerning HL among farmers and agricultural workers, particularly in developing countries, with a specific emphasis on rural areas. Notably, there is a lack of studies on HL among populations in South America and Europe (10). Given that approximately 78% of the world’s poorest people reside in rural settings and depend largely on agriculture settings (11, 12). The available studies indicated that HL levels among agricultural workers and rural residents tend to be lower compared to other population groups (10, 13, 14). Studies conducted in developing countries have also identified significant differences in HL between rural and urban populations, in contrast to studies conducted in developed countries (10). In Thailand, previous studies currently available also investigated HL in the general population and in older people (7, 15). Despite several studies taking place in farmers in Thailand, these only investigated in specific geographical areas in north and North-East Thailand (16, 17). Additionally, data regarding agricultural and protective behavior factors related to HL among farmers of all regions in Thailand are limited.

The agricultural sector in Thailand covers 6.4 million households and employs approximately 30% of the total labor force. However, the agricultural sector accounted for only 8.8% of gross domestic product (GDP) in 2022 (18, 19). Even though it is only a small proportion of GDP, agriculture is crucial to Thai economics because the vast majority of the population still depend on it for their livelihood. The agricultural sector is currently facing several problems, including poverty, aging, land ownership, small-scale farming, and a limited farming portfolio (19). In addition, they also face a problem of adverse health effects from pesticide use (20). Thai farmers encounter a pressing issue concerning the misuse and overuse of pesticides. Approximately 80% of the pesticides applied exceed the socially optimal quantity (21). The external costs associated with pesticide use are considerable, averaging USD 27.1 per hectare of agricultural land (22). Moreover, pesticide prices would have minimal impact on curbing this overuse trend. Given these challenges, there is an urgent need for supportive measures aimed at altering on-farm practices and promoting education on alternative pest management strategies (22, 23). In recent years, the Thai government has promoted the use of technology and innovation to enhance the quality of the agricultural products and to support farmers (24). However, there is a lack of knowledge in alternative pest management and new technology in agriculture in Thai farmers due to their low levels of literacy (19, 20, 25). As a result, farmers and agricultural workers are especially vulnerable to adverse health effects from pesticide exposure due to their HL. This study aims to investigate HL among farmers in four main regions of Thailand, examining socio-demographics, agricultural, and personal protective behavior factors in relation to their HL. The expected outcomes of this study are identification of the most vulnerable target population and areas to ensure that strategies and interventions should be focused on the area of highest need to enhance HL and reduce health disparities in Thailand.

## 2 Methods

### 2.1 Setting and participants

Between January and July 2023, a study cross-sectional in design was conducted to survey HL among farmers in four regions of Thailand. The study’s participants were farmers who used pesticides in agriculture and lived in four regions of Thailand, including Central, North, North-East, and South regions. The total agricultural population in Thailand’s four regions was 6,744,856, with 1,249,490 people living in the Central region, 864,400 in the North, 3,503,763 in the North-East, and 1,127,203 in the South (26). The EpiInfo program was used to calculate sample size, with an expected frequency of 40%, 2% confidence limits, and 99% confidence interval level, therefore 3,979 was the minimum sample size needed. Multi-stage stratified random sampling was employed, dividing the farmer populations into four strata based on the regions of Thailand. The study selected 31 provinces in Thailand, which are predominantly agricultural areas, as its focus. These provinces included seven from the Central region, four from the North, 14 from the North-East, and six from the South. Seven provinces from the Central region, including Nakhon Sawan, Lopburi, Nakhon Prathom, Saraburi, Sing Buri, Ayutthaya, and Chai Nat provinces, were chosen to represent the central region, representing 13.7% of the Central. Four provinces from the North region, including Lam Pang, Phayao, Chiang Mai, and Chiang Rai provinces, were designated as representative areas of the North, representing 23.5% of the North. Fourteen provinces from the North-East region, including Ubon Ratchathani, Sisaket, Surin, Buriram, Nakhon Ratchasima, Udon Thani, Mukdahan, Kalasin, Amnat Charoen, Roi Et, Yasothon, Chaiyaphum, Nakhon Phanom, and Khon Kaen provinces were chosen to represent the North-East, representing 70% of the North-East. Six provinces from the South region, including Pattani, Yala, Surat Thani, Songkhla, Narathiwat, and Nakhon Si Thammarat provinces were identified as representative areas of the South, representing 42.9% of the North-East. Samples were systematically drawn from agricultural and rural communities within each province. Population proportional sampling was used, ensuring that the sample size for each stratum was directly proportional to the population size of that specific stratum. The actual sample sizes were 746 from the Central region, 586 from the North, 2,065 from the North-East, and 638 from the South.

### 2.2 Interviews

Due to the illiteracy of the majority of Thai farmers face-to-face interviews were conducted. The participants were interviewed with specifically trained interviewers. This study involved researchers located across all regions of Thailand. To reduce cultural and language differences in each region of Thailand, researchers in each region provided training to interviewers on purpose of the study, questions in the questionnaire, questions and scales in HLS-EU-Q47, and the need for professional and consistent performance by the interviewers. These interviewers were either healthcare personnels or bachelor students in health sciences who resided in the study areas and were fluent in the local languages spoken in those areas.

The questions on the interview form were divided into four sections, including socio-demographic characteristics, agricultural information, personal protective behavior, and HL. Socio-demographic data included age (years), gender (male or female), marital status (single, married, or divorced or widow), education level (elementary school or lower, secondary school, or Bachelor degree or higher), monthly income (≤250 US Dollars or > 250 US Dollars), co-morbidity (yes or no), number of family member (persons), cigarette smoker (yes or no), alcohol consumption (yes or no), and acting as a health volunteer in the village (yes or no). Agricultural information included planting area (acres), planting status (planting in own area or as a hiree), working hours on farm [2–4 h (h)/day or > 4 h./day], type of crop planting (rice, corn, para rubber, cassava, sugarcane, palm, vegetable, fruit, flower, or other), task on the farm (mixing pesticides, spraying pesticides, or harvesting crops), type of pesticide use (herbicide, insecticide, fungicide, molluscicide, rodenticide, or nematocide), and source of pesticide information (pesticide merchants, neighbors, government officers, online multimedia, television, posters/brochures, or radio). Regarding personal protective behavior, the questions asked about personal protective equipment (PPE) use during pesticide application including wearing of a long-sleeved shirt, long-sleeved trousers, hat, gloves, goggles, mask, boots, and rubber apron.

HLS-EU-Q47 was used to measure HL (5). The HLS-EU-Q47 includes 47 items, each item being rated on a four-point Likert rating scale, specifically: very difficult (1), difficult (2), easy (3), and very easy (4). The conceptual model of the HLS-EU-Q47 distinguishes between three contexts of HL (health care, disease prevention, and health promotion) and four competencies to deal with information relative to health (access, understand, appraise, and apply). These three contexts and the four competencies were then used to create seven subdomains for measuring HL. The seven subdomains were as follows: Subdomain1: health care (16 items); Subdomain 2: disease prevention (15 items); Subdomain 3: health promotion (16 items); Subdomain 4: access/obtain information relevant to health (13 items); Subdomain 5: understand information relevant to health (11 items); Subdomain 6: process/appraise information relevant to health (12 items); and Subdomain 7: apply/use information relevant to health (11 items). The HL index was standardized to a scale of 0–50 using the formula:


$$HL\;\mathrm{Index}=\left(\mathrm{mean}-1\right)\times \left(50/3\right)$$

where,

HL index was the HL index calculated.

Mean was the mean of HL for each index.

Health literacy (HL) was classified into four levels, specifically: inadequate HL (scores of 0–25), likely problematic HL (>25–33 scores), likely sufficient HL (>33–42 scores), and excellent HL (>42–50 scores). To detect vulnerable groups, inadequate HL and likely problematic HL were combined and called limited HL (scores of 0–33).

The HLS-EU-Q47 was translated from English into Thai language using forward-only translation by three native Thai researchers who were fluent in English (RS, JJ, and AK). To reduce cultural and language differences in each region, The Thai version of The HLS-EU-Q47 were verified and edited by the seven researches from all regions of Thailand (EC, CS, PT, AT, BS, NSa, and TS). Validity was tested by three experts (ST, NSi, and AL). Pilot testing was performed in 10 farmers from all regions. Minor wording, typographical errors, and grammar were corrected, and the Thai final version was prepared by RS. The index of congruence (IOC) score was >0.5. Internal consistency was tested using Cronbach’s alpha coefficient, and the coefficient of The HLS-EU-Q47 in the Thai version was 0.876 therefore the questionnaire was a reliable tool.

### 2.3 Ethical approval

The study was approved by the Research Ethics Committee of the Faculty of Medicine, Chiang Mai University, Thailand (No. 419/2022). Prior to the interviews, the participants were required to complete a written informed consent form. The participants were informed regarding the confidentiality and data protection protocols in place. No photographic or video recordings of participants were made. Furthermore, personal identifiers such as names, addresses, and telephone numbers were not solicited in the interview documentation. Moreover, access to the dataset files is restricted solely to the researcher team, safeguarded by password protection.

### 2.4 Statistical analysis

**Figure 1:**
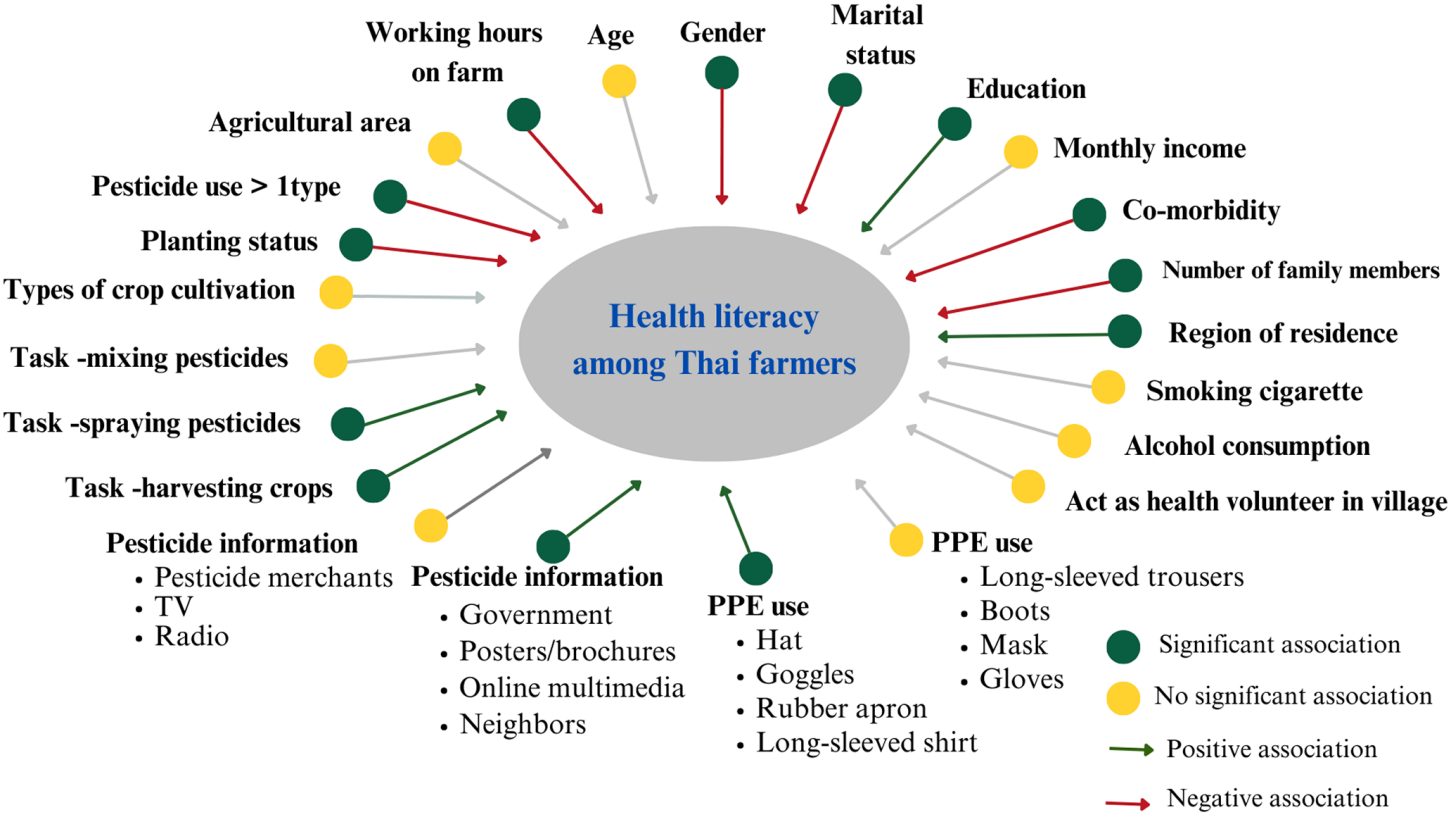
Factors associated with health literacy among Thai farmers.

Descriptive statistics, including frequency (n), percentage (%), mean, and standard deviation (SD) were used. Univariable analysis, including independent *t*-test, and one-way ANOVA were used to investigate the variables associated with the HL score. The variables that had a significant (*p* value<0.05) from the univariable analysis were included in the multivariable model. Therefore, 29 variables from the univariable analysis that were significant were included into the model of multiple linear regression analysis (Figure 1).The multiple linear regression analysis was used to investigate the factors associated with HL score. Beta, standard error (SE) and 95% confidence intervals (95%CI) are presented.

## 3 Results

### 3.1 Socio-demographics and agricultural information among farmers

The mean age of farmers was 52.5 ± 10.8 years, and 45.6% of them were male. Approximately 77% of farmers were married, 63% had elementary school or lower levels of education, and 66.8% had a monthly income ≤250 US Dollars. A total of 19% of the farmers smoked cigarettes while 31.6% drank alcohol. Approximately 29.6% of farmers had a co-morbidity, and 27.3% were health volunteers in their village (Table 1).

**Table 1:** Socio-demographic characteristics among farmers classified by regions of Thailand.

Parameters		Total (*N* = 4,035)	North-East (*n* = 2,065)	Central (*n* = 746)	South (*n* = 638)	North (*n* = 586)
Age (years old), mean ± SD.		52.5 ± 10.8	52.9 ± 11.1	52.6 ± 10.9	48.7 ± 10.9	55.0 ± 8.1
Gender, *n* (%)	Male	1841 (45.6)	1,019 (49.3)	287 (38.5)	200 (31.3)	335 (57.2)
	Female	2,194 (54.4)	1,046 (50.7)	459 (61.5)	438 (68.7)	251 (42.8)
Marital status, *n* (%)	Single	570 (14.1)	318 (15.4)	122 (16.4)	77 (12.1)	53 (9.0)
	Married	3,116 (77.2)	1,613 (78.1)	522 (70.0)	539 (84.5)	442 (75.4)
	Divorced/widow	349 (8.6)	134 (6.5)	102 (13.7)	22 (3.4)	91 (15.5)
Education level, *n* (%)	Elementary school or lower	2,542 (63.0)	1,257 (60.9)	387 (51.9)	375 (58.8)	523 (89.2)
	Secondary school	1,202 (29.8)	676 (32.7)	277 (37.1)	190 (29.8)	59 (10.1)
	Bachelor degree or higher	291 (7.2)	132 (6.4)	82 (11.1)	73 (11.4)	4 (0.7)
Monthly income, *n* (%)	≤250 US Dollars	2,695 (66.8)	1,621 (78.5)	381 (51.1)	264 (41.4)	429 (73.2)
	>250 US Dollars	1,340 (33.2)	444 (21.5)	365 (48.9)	374 (58.6)	157 (26.8)
Co-morbidity, *n* (%)		1,194 (29.6)	432 (20.9)	381 (51.1)	200 (31.3)	181 (30.9)
Family members (persons), mean ± SD		4 ± 2	4 ± 2	4 ± 2	4 ± 2	3 ± 1
Smoking cigarette, *n* (%)		767 (19.0)	116 (15.5)	403 (19.5)	84 (13.2)	164 (28.0)
Alcohol consumption, *n* (%)		1,275 (31.6)	692 (33.5)	204 (27.3)	36 (5.6)	343 (58.5)
Acting as a health volunteer in village, *n* (%)		1,102 (27.3)	431 (20.9)	369 (49.5)	129 (20.2)	173 (29.5)

n, frequency; %, percentage; SD, Standard deviation.

Regarding agricultural information, 60.6% of farmers had a planting area ≤ 4 acres, 78.2% planted in their own area, and 54.4% worked on the farm 2–4 h/day. The most frequently planted crops were rice (76.7%), followed by vegetables (34.2%) and then fruit (16.0%). The most common pesticides used were herbicides (77.3%), followed by insecticides (68.8%) and fungicides (54.5%). About 75.4% of the farmers used more than one type of pesticide on their farm. The farmers’ tasks included mixing pesticide (62.1%), spraying pesticides (61.6%), and harvesting crops (65.9%). Pesticide merchants were the most frequent sources of information (62.3%), followed by neighbors (53.3%) and government officials (33.4%) (Table 2).

**Table 2:** Agricultural information among farmers classified by regions of Thailand.

Parameters		Total (*N* = 4,035)	North-east (*n* = 2,065)	Central (*n* = 746)	South (*n* = 638)	North (*n* = 586)
Agricultural area (acres), *n* (%)	≤ 4 acres	2,446 (60.6)	1,289 (62.4)	162 (21.7)	514 (80.6)	481 (82.1)
	> 4 acres	1,589 (39.4)	776 (37.6)	584 (78.3)	124 (19.4)	105 (17.9)
Planting status, *n* (%)	Planting in own area	3,157 (78.2)	1,903 (92.2)	453 (60.7)	552 (86.5)	249 (42.5)
	Hiree	878 (21.8)	162 (7.8)	293 (39.3)	86 (13.5)	337 (57.5)
Working hours on farm, *n* (%)	2–4 h./day	2,196 (54.4)	1,377 (66.7)	279 (37.4)	448 (70.2)	92 (15.7)
	>4 h./day	1,839 (45.6)	688 (33.3)	467 (62.6)	190 (29.8)	494 (84.3)
Type of crop planting, *n* (%)	Rice	3,094 (76.7)	1,895 (91.8)	581 (77.9)	79 (12.4)	539 (92.0)
	Vegetable	1,379 (34.2)	792 (38.4)	306 (41.0)	188 (29.5)	93 (15.9)
	Fruit	646 (16.0)	236 (11.4)	200 (26.8)	177 (27.7)	33 (5.6)
	Para rubber	558 (13.8)	129 (6.2)	4 (0.5)	409 (64.1)	16 (2.7)
	Corn	460 (11.4)	131 (6.3)	196 (26.3)	53 (8.3)	80 (13.7)
	Cassava	430 (10.7)	296 (14.3)	48 (6.4)	28 (4.4)	58 (9.9)
	Flower	351 (8.7)	204 (9.9)	122 (16.4)	24 (3.8)	1 (0.2)
	Sugarcane	278 (6.9)	176 (8.5)	78 (10.5)	24 (3.8)	0 (0)
	Palm	141 (3.5)	13 (0.6)	1 (0.1)	126 (19.7)	1 (0.2)
	other	208 (5.2)	83 (4.0)	32 (4.3)	74 (11.6)	19 (3.2)
Task on farm, *n* (%)	Mixing pesticides	2,505 (62.1)	1,243 (60.2)	527 (70.6)	322 (50.5)	413 (70.5)
	Spraying pesticides	2,486 (61.6)	1,196 (57.9)	531 (71.2)	324 (50.8)	435 (74.2)
	Harvesting crops	2,660 (65.9)	1,317 (63.8)	474 (63.5)	341 (53.4)	528 (90.1)
Type of pesticides use, *n* (%)	Herbicides	3,120 (77.3)	1,503 (72.8)	652 (81.4)	403 (63.2)	562 (95.9)
	Insecticides	2,775 (68.8)	1,392 (67.4)	644 (86.3)	278 (43.6)	461 (78.7)
	Fungicides	2,198 (54.5)	1,1,53 (55.8)	554 (74.3)	150 (23.5)	341 (58.2)
	Nematocides	1,986 (49.2)	998 (48.3)	563 (75.5)	130 (20.4)	295 (50.3)
	Molluscicides	1,580 (39.2)	876 (42.4)	383 (51.3)	40 (6.3)	281 (48.0)
	Rodenticides	1,471 (36.5)	792 (38.4)	418 (56.0)	95 (14.9)	166 (28.3)
Number of pesticides used, *n* (%)	1 type	993 (24.6)	533 (25.8)	78 (10.5)	340 (53.3)	42 (7.2)
	> 1 type	3,042 (75.4)	1,532 (74.2)	668 (89.5)	298 (46.7)	544 (92.8)
Source of pesticide information, *n* (%)	Pesticide merchants	2,514 (62.3)	1,219 (59.0)	583 (78.2)	296 (46.4)	416 (71.0)
	Neighbors	2,151 (53.3)	1,157 (56.0)	393 (52.7)	391 (61.3)	210 (35.8)
	Government officers	1,346 (33.4)	861 (41.7)	279 (37.4)	108 (16.9)	98 (16.7)
	Online multimedia	961 (23.8)	351 (17.0)	325 (43.6)	210 (32.9)	75 (12.8)
	Television	833 (20.6)	324 (15.7)	231 (31.0)	207 (32.4)	71 (12.1)
	Posters/brochures	661 (16.4)	392 (19.0)	133 (17.8)	41 (6.4)	95 (16.2)
	Radio	576 (14.3)	258 (12.5)	214 (28.7)	60 (9.4)	44 (7.5)

### 3.2 PPE use during pesticide application among farmers

**Figure 2:**
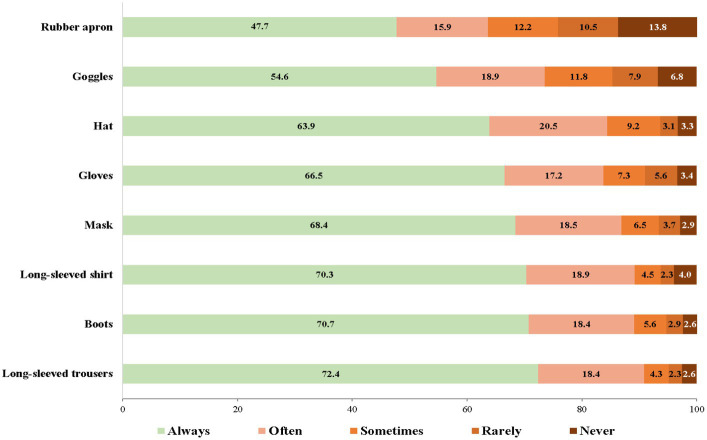
PPE use during pesticide application.

The PPE that farmers always wore while applying pesticides were long-sleeved trousers (72.4%), followed by boots (70.7%), and a long-sleeved shirt (70.3%), while the PPE that farmers never wore while applying pesticides were a rubber apron (13.8%), goggles (6.8%), and a hat (3.3%) (Figure 2).

### 3.3 HL among farmers

**Figure 3:**
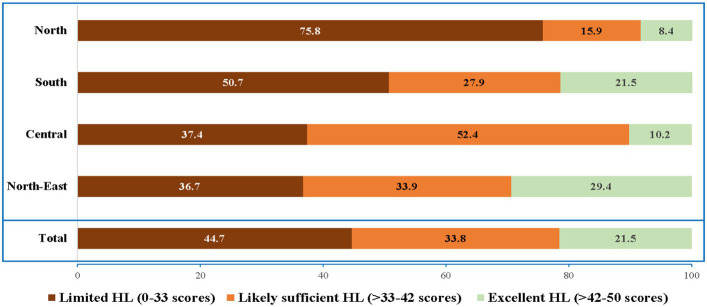
Health literacy among farmers, classified by regions of Thailand.

The average HL score among Thai farmers was 34.7 ± 8.7. The North-east region had the highest mean HL score (36.8 ± 8.9), followed by Central (34.6 ± 5.9), South (33.5 ± 9.3), and North (29.0 ± 6.9). Considering the subdomains of HL scores, HL of all subdomains for the North-east region had the highest HL scores, while HL of all subdomains in the North had the lowest HL scores (Table 3). To detect groups most vulnerable to low levels of HL in each region, the results were analyzed and showed that farmers in the North region had the highest frequency of limited HL (75.8%), followed by the South (50.7%), the Central (37.4%), and the North-East (36.7%) (Figure 3).

**Table 3:** HL scores among farmers, classified by regions of Thailand.

Parameters	Mean ± SD.					
	Total (*N* = 4,035)	North-east (*n* = 2,065)^a^	Central (*n* = 746)^b^	South (*n* = 638)^c^	North (*n* = 586)^d^	*p* value
Total scores HL	34.7 ± 8.7	36.8 ± 8.9	34.6 ± 5.9	33.5 ± 9.3	29.0 ± 6.9	<0.001^ab,ac,ad,bc,bd,cd^
Sub-domain 1: Access/Obtain information relevant health	33.8 ± 9.4	35.6 ± 9.5	34.7 ± 6.5	33.3 ± 9.4	26.5 ± 8.3	<0.001 ^ab,ac,ad,bc,bd,cd^
Sub-domain 2: Understand information relevant to health	36.3 ± 8.9	38.5 ± 9.1	36.2 ± 6.4	34.0 ± 9.5	31.2 ± 7.7	<0.001 ^ab,ac,ad,bc,bd,cd^
Sub-domain 3: Process/appraise information relevant to health	33.7 ± 9.3	35.9 ± 9.5	32.2 ± 7.8	33.5 ± 9.4	28.0 ± 7.2	<0.001 ^ab,ac,ad,bc,bd,cd^
Sub-domain 4: Apply/use information relevant to health	35.4 ± 8.8	37.3 ± 9.2	35.5 ± 6.2	33.3 ± 9.8	30.9 ± 6.3	<0.001 ^ab,ac,ad,bc,bd,cd^
Sub-domain 5: Health care	35.0 ± 8.9	36.9 ± 9.4	35.5 ± 6.4	33.6 ± 9.6	29.5 ± 6.7	<0.001 ^ab,ac,ad,bc,bd,cd^
Sub-domain 6: Disease prevention	33.7 ± 9.4	35.9 ± 9.7	32.5 ± 7.4	32.9 ± 9.9	28.2 ± 7.2	<0.001 ^ab,ac,ad,bc,bd,cd^
Sub-domain 7: Health promotion	35.4 ± 9.2	37.4 ± 9.3	35.6 ± 6.7	33.9 ± 9.5	29.3 ± 9.3	<0.001 ^ab,ac,ad,bc,bd,cd^

The different letters refer to significant differences.

### 3.4 Factors related to HL in farmers

Univariable analysis showing the factors associated with HL scores among Thai farmers are presented in Table 4, and multiple linear regression analysis are presented in Table 5.

**Table 4:** Univariable analysis for investigating factors associated with HL among Thai farmers.

Parameters			HL scores (mean ± SD)	*p* value
Gender	Male		34.4 ± 8.8	0.031^*^
	Female		35.0 ± 8.5	
Status	Single^a^		36.6 ± 9.4	<0.001^** ab,ac^
	Married^b^		34.5 ± 8.6	
	Widowed/divorced/^c^		34.1 ± 7.7	
Education level	Elementary school or lower^a^		33.9 ± 8.7	<0.001^**ab,ac^
	Secondary school^b^		35.9 ± 8.6	
	Bachelor degree or higher^c^		36.3 ± 7.7	
Monthly income	≤250 US Dollars		34.9 ± 8.5	0.181
	>250 US Dollars		34.5 ± 8.9	
Co-morbidity	Yes		34.0 ± 7.7	<0.001^**^
	No		35.0 ± 9.0	
Smoking cigarette	Yes		33.5 ± 8.3	<0.001^**^
	No		35.0 ± 8.7	
Alcohol consumption	Yes		33.6 ± 8.1	<0.001^**^
	No		35.2 ± 8.9	
Acting as a health volunteer	Yes		34.9 ± 7.9	0.472
	No		34.7 ± 8.9	
Agricultural area	≤ 4 acres		34.2 ± 9.1	<0.001^**^
	> 4 acres		35.6 ± 7.9	
Planting status	Planting in own area		35.7 ± 8.7	<0.001^**^
	Hiring		31.3 ± 7.6	
Working hours on farm	2–4 h./day		36.4 ± 9.0	<0.001^**^
	>4 h./day		32.8 ± 7.8	
Tasks on farm	Mixing pesticides	Yes	35.2 + 8.9	<0.001^**^
		No	33.9 ± 8.2	
	Spraying pesticides	Yes	35.4 ± 9.0	<0.001^**^
		No	33.6 ± 7.9	
	Harvesting crop products	Yes	34.9 ± 9.1	0.028^*^
		No	34.3 ± 7.9	
Number of pesticides used	1 type		34.5 ± 8.4	0.277
	>1 type		34.8 ± 8.8	
Sources of pesticide information	Neighbors	Yes	35.6 ± 8.7	<0.001^**^
		No	33.8 ± 8.6	
	Pesticide merchants	Yes	35.2 ± 8.8	<0.001^**^
		No	33.9 ± 8.5	
	Government officers	Yes	37.4 ± 8.8	<0.001^**^
		No	33.4 ± 8.3	
	Posters/brochures	Yes	36.0 ± 7.2	<0.001^**^
		No	34.5 ± 8.9	
	Online multimedia	Yes	35.5 ± 7.2	<0.001^**^
		No	34.5 ± 9.1	
	Radio	Yes	35.1 ± 7.2	0.176
		No	34.7 ± 8.9	
	Television	Yes	34.2 ± 6.2	0.052
		No	34.9 ± 9.2	

^*^p value < 0.05; ^**^p value < 0.01.

The different letters refer to significant differences.

**Table 5:** Multiple linear regression analysis for investigating factors associated with HL among Thai farmers.

	Beta	SE.	95% CI		*p* value
			Lower bound	Upper bound	
Age	0.020	0.013	−0.005	0.046	0.118
Gender	−0.698	0.284	−1.255	−0.141	0.014^*^
Region	1.503	0.149	1.210	1.796	<0.001^**^
Marital status	−0.964	0.262	−1.479	−0.450	<0.001^**^
Education level	0.602	0.218	0.175	1.029	0.006^**^
Co-morbidity	−0.791	0.273	−1.326	−0.256	0.004^**^
Number of family members	−0.345	0.071	−0.484	−0.205	<0.001^**^
Smoking cigarette	−0.374	0.352	−1.065	0.317	0.288
Alcohol consumption	−0.547	0.300	−1.134	0.041	0.068
Planting status	−3.125	0.320	−3.752	−2.498	<0.001^**^
Agricultural area	0.053	0.267	−0.470	0.576	0.842
Working hours on farm	−2.451	0.261	−2.964	−1.939	<0.001^**^
Mixing pesticides	0.075	0.393	−0.695	0.845	0.848
Spraying pesticides	2.449	0.389	1.686	3.211	<0.001^**^
Harvesting crops	0.868	0.275	0.329	1.407	0.002^**^
Pesticide use of more than 1 type	−1.502	0.326	−2.142	−0.862	<0.001^**^
Pesticide information-Government officers	2.009	0.269	1.482	2.537	<0.001^**^
Pesticide information-Posters/brochures	1.465	0.326	0.826	2.103	<0.001^**^
Pesticide information-Online multimedia	0.969	0.292	0.396	1.542	0.001^**^
Pesticide information-Neighbors	0.949	0.246	0.467	1.432	<0.001^**^
Pesticide information-Pesticide merchants	0.424	0.256	−0.077	0.926	0.097
PPE use-Hat	1.302	0.203	0.903	1.700	<0.001^**^
PPE use-Goggles	0.942	0.142	0.663	1.221	<0.001^**^
PPE use-Rubber apron	0.487	0.110	0.270	0.703	<0.001^**^
PPE use-Long-sleeved shirt	0.709	0.196	0.325	1.094	<0.001^**^
PPE use-Long-sleeved trousers	−0.115	0.283	−0.670	0.440	0.685
PPE use-Boots	0.025	0.268	−0.499	0.550	0.925
PPE use-Mask	−0.260	0.226	−0.703	0.185	0.250
PPE use-Gloves	−0.269	0.200	−0.662	0.123	0.179

^*^p value < 0.05; ^**^p value < 0.01.

Multiple linear regression analysis found that socio-demographic factors associated with HL scores included gender, region, marital status, level of education, co-morbidity, and number of family member.

Agricultural factors that were associated with HL scores included planting status, working hours on the farm, spraying pesticides, harvesting crops, use of >1 type of pesticide, access information from government officers, access information from posters/brochures, access information from online multimedia, and access information from neighbors.

Personal protective factors that were associated with HL scores included wearing a hat, wearing goggles, wearing a rubber apron, and wearing a long-sleeved shirt.

## 4 Discussion

According to our findings, Thai farmers had a mean HL score of 34.7 ± 8.7. When the HL was compared to other studies that used the same HLS-EU-Q47 for measuring general HL, the farmers’ HL scores in our results were comparable to those of farmers in a prior study conducted in northern Thailand, where the mean HL score was 34.98 (17). A study by Sørensen et al. (9) investigated HL in eight European countries, and found the average HL scores were 31.95 ± 7.63 in Austria, 30.50 ± 9.17 in Bulgaria, 34.49 ± 7.87 in Germany, 33.57 ± 8.48 in Greece, 35.16 ± 7.79 in Ireland, 37.06 ± 6.40 in Netherlands, 34.45 ± 7.98 in Poland, and 32.88 ± 6.10 in Poland. A study by Duong et al. (8) also investigated HL in six Asian countries, and found the average HL scores were 31.4 ± 5.8 in Indonesia, 31.6 ± 9.3 in Kazakhstan, 32.9 ± 7.2 in Malaysia, 31.3 ± 8.7 in Myanmar, 34.4 ± 6.6 in Taiwan, and 29.6 ± 9.1 for Vietnam. However, these two investigations were conducted in general populations. As a consequence, the interpretation of the data might not be comparable. Considering with subdomains of HL, our revealed that Thai farmers obtained the lowest scores of HL in tow sub-domains: Sub-domain 2: process/appraise information relevant to health Disease prevention and Sub-domain 6: disease prevention. These findings suggest that the farmers had low ability to interpret and evaluate information on risk factors for health. Therefore, it is crucial for the government and relevant organizations to prioritize evidence-based strategies and interventions aimed at improving farmers’ ability to interpret and evaluate information regarding health risk factors, particularly those associated with pesticide use.

Socio-demographic factors were crucial in relation to HL. According to our findings, socio-demographic factors that were related to the HL scores of farmers included gender, marital status, education level, co-morbidity, and family members. Farmers who were female, single, had a high level of education, had a small size of family members, and had no co-morbid conditions were likely to have a high level of HL.Our findings were consistent with a study by Chakraverty et al. (27) which found clear evidence that women typically had a higher level of HL than men. They also mentioned that future research should consider men and women separately to ensure reliable results. A study by Sun et al. (28) identified gender differences in factors associated with the HL in older patients with chronic diseases. They suggested that HL in men was associated with education background, number of dependents, monthly income, duration of chronic disease, and self-efficacy; while HL in women was associated with age, education background, monthly income, duration and treatment of chronic diseases.

Considering education level, our results were also in line with a study by Jansen et al. (29) which suggested that attainment of higher levels of education was associated with higher scores of HL in aspects of appraisal of health information and the healthcare system. People with low levels of education had difficulty understanding and assessing health-related information. As a result, they were unable to successfully communicate with the health care system. On the other hand, people with high education found it easier to access, comprehend, and assess knowledge of health–related issues (30, 31).Walters et al. (32) suggested that educational interventions could enhance HL and health-promoting behaviors. Face-to-face education and non-print media (such as video, images, etc.) were the most effective ways to transfer health messages to people with low levels of HL (33). The government, media, and relevant organizations should collaborate effectively to provide HL services and focus on fairness of health education (34).

Regarding marital status, our findings found that farmers who were single or married had higher HL scores than widows or divorcees. It is possible that individuals who were widowed or divorced had a lack of motivation to attend to things related to health education due to psychological, spiritual, and economical issues (35). Another possibility is that other family members may support and compensate individuals to achieve health-related tasks (36, 37). Co-morbid conditions were also associated with HL in both general populations and patient groups (37, 38). Therefore, our findings suggested that in order to improve the HL of farmers, target populations should be prioritized when it comes to strategies and interventions, specifically including men, those who are widowed or divorced, those with low levels of education, and those who have co-morbidities.

Farmers’ residential region also affected their level of HL. Our findings found that farmers in the North region of Thailand had the lowest level of HL, while those in the North-East had the highest HL. Importantly, farmers in the North region had the highest frequency of limited HL (75.8%). It is possible that this was because the majority of farmers in the North region (89.2%) only had completed an elementary school or lower, while in the North-East there was the largest percentage of farmers with a bachelor degrees or above (7.2%) when compared to other regions. Therefore, strategies and interventions should be focused on the farming population in the North region to improve health equity in Thailand.

Regarding agricultural aspects, our results found that farmers who planted on their own farm, worked on the farm 2–4 h/day, sprayed pesticides, harvested crops, and applied only one type of pesticide on their farm were more likely to have a higher level of HL. These findings were consistent with a study by Montgomery et al. (17) which mentioned that length of time farming, and no chemical use in farming were associated with high HL in farmers. One previous piece of research in Thailand also suggested that functional literacy was significantly associated with pesticide use behaviors among sweet corn farmers (16). Our results also found that farmers who got pesticide information from government officers, posters/brochures, online multimedia, and neighbors were also likely to have high levels of HL. Interestingly, the information from government officers showed the strongest association with HL, followed by posters/brochures. In contrast, the study by Li et al. (3) mentioned that the four main sources of health information for older adults in China were healthcare practitioners, neighbors, newspapers, and television. It is possible that the different population groups might affect their health information sources. It is also possible that sources of health information for various population groups varied depending on their ages and region of residence. The findings from our study suggested that in-person education from government officers and posters/brochures were the most efficient means of conveying health-related information to farmers. Improvement of HL through both interpersonal and mass communication means should be simultaneously implemented (3).

Regarding personal protective behavior, PPE use during pesticide application was also a crucial factor related to the HL of farmers. Our results found that farmers who wore a hat, goggles, a rubber apron, and a long-sleeved shirt were likely to have high HL. Available previous studies clearly mention that wearing of PPE and prevention practices against pesticide exposure were the main factors influencing the HL of farmers (39, 40). A systematic review by Sapbamrer and Thammachai (41) concluded that the most basic PPE worn among pesticide handlers in all regions of the world was a long-sleeved shirt (66.1%), long-sleeved trousers (71.1%), and a hat (47.3%), while the lowest basic PPE worn was an apron (8.6%), goggles (24.3%), gloves (40.5%), boots (42.3%), and a mask (43.2%). In Thailand, farmers usually wore everyday available clothing, a fabric hat, and fabric mask to protect themselves from exposure to pesticides. However, they hardly ever used goggles, respirators, and rubber aprons during pesticide application. The main reason included tropical climatic weather conditions, discomfort while working, poverty, lack of availability of PPE, and the expense of PPE (25). A systematic review by Sapbamrer and Thammachai (41) also mentioned that access to extension services, training programs, information about pesticides, and farm organization are all crucial determinants associated with PPE use. Education interventions to improve safety and HL for farmers with a variety of approaches that take into account cultural aspects, individual factors, and community involvement had the greatest outcomes with regard to changing the behavior of farmers (42, 43). Several approaches have been used in educational interventions such as training, movies, newsletters, community fairs, games, and community engagement in program design (42). It is evident that a life-long education program regarding safety and health-related concerns with several approaches could enhance the HL of farmers and sustainably improve pesticide safety behavior.

Previous research conducted in Thailand highlighted a significant gap in the knowledge of alternative pest management and modern agricultural technology among Thai farmers, due to their limited literacy levels (19, 20, 25). Integrating biological control methods is crucial within the framework of integrated pest management (IPM), aimed at reducing use of pesticides and chemicals. The adoption of IPM not only yields immediate benefits but also contributes to developmental benefits across technical, social, and political domains (44). However, the adoption and implementation of IPM and biological control worldwide were rather slow (45). Furthermore, a meta-analysis conducted by Tamburini et al. (46) strongly suggests that agricultural diversification practices can significantly enhance various ecosystem such as biodiversity, pollination, pest control, nutrient cycling, soil fertility, and water regulation, all while maintaining crop yields. Consequently, there is an urgent need for participatory training programs to promote the adoption of IPM and agricultural diversification practices, fostering the sustainable development of agro-ecosystems (44).

The data for the present study were gathered from 31 provinces, accounting for 40.3% of all provinces, and from four main regions of Thailand. As a result, the findings can be generalized to all Thai farmers nationwide. Additionally, the findings regarding HL can be provided visually in regional hot spots, and further target actions appropriately to reduce limited HL and health disparities. However, limitations to our study exist. First, the data on pesticides used could only be collected in terms of the types of pesticides, not their active components or common names, due to the low literacy levels of the farmers. Second, recall-bias for agricultural information over the past 3 months might have occurred, limiting accuracy of data. Third, the presence of language and cultural disparities across various regions in Thailand may serve as confounding variables that impact the interpretation of study findings. Fourth, a cross-sectional study design cannot explore relationships between cause and effect. These limitations should be taken into account for further investigations.

## 5 Conclusion

The findings of this study indicate that farmers in the North region of Thailand had the highest frequency of limited HL. Socio-demographic factors associated with the HL of farmers included gender, marital status, education level, co-morbidity, and the number of family members. Agricultural factors associated with the HL of farmers included planting status, working hours on the farm, task of the farm, number of pesticides used, and PPE use during pesticide application. Therefore, the recommendations from our study are that strategies and interventions to enhance HL in farmers should be focused on the target populations which include men, widows or divorcees, those with low levels of education, those who have co-morbidities, and those who apply pesticides of more than 1 type, and improper PPE use. The North region of Thailand should be the primary emphasis of the target area to improve health equity in Thailand. The most effective way to transfer knowledge about safety and health-related issues to farmers is via a lifelong education program using several approaches, along with posters and brochures. Improvements in HL and health equity need to be urgently addressed by the government and relevant organizations to develop and launch appropriate health promotion strategies and interventions. Importantly, the government and relevant organizations need to focus on evidence-based strategies and interventions aimed at improving farmers’ ability to interpret and evaluate information regarding health risk factors, particularly those associated with pesticide use. Interventions aimed at enhancing farmers’ capacity to interpret and evaluate information concerning the safe uses of pesticides and preventive measures from pesticides are urgently needed. These efforts could enhance the HL of farmers and improve pesticide safety behavior sustainably.

Further research involving longitudinal study design could provide valuable insights into addressing these issues. Additionally, intervention studies aimed at enhancing farmers’ HL, particularly their ability to interpret and evaluate risk factors information for health are warranted. Specifically within the context of pesticides, interventions focusing on enhancing farmers’ capacity to interpret and evaluate information concerning the safe uses of pesticides and preventive measures from pesticides are also warranted. Prioritizing intervention studies targeting the farming population in the North region could contribute significantly to improving health equity in Thailand.

## Data availability statement

The original contributions presented in the study are included in the article/supplementary material, further inquiries can be directed to the corresponding author.

## Ethics statement

The studies involving humans were approved by the Research Ethics Committee of the Faculty of Medicine, Chiang Mai University, Thailand (No. 419/2022). The studies were conducted in accordance with the local legislation and institutional requirements. The participants provided their written informed consent to participate in this study.

## Author contributions

RS: Conceptualization, Data curation, Formal Analysis, Funding acquisition, Investigation, Methodology, Project administration, Resources, Software, Supervision, Validation, Visualization, Writing – original draft, Writing – review & editing. NaS: Formal Analysis, Investigation, Methodology, Writing – review & editing. ST: Investigation, Methodology, Writing – review & editing. EC: Investigation, Methodology, Writing – review & editing. CS: Investigation, Methodology, Writing – review & editing. AL-L: Investigation, Methodology, Writing – review & editing. PT: Investigation, Methodology, Writing – review & editing. AT: Formal Analysis, Writing – review & editing. BS: Formal Analysis, Writing – review & editing. NoS: Formal Analysis, Writing – review & editing. AK: Formal Analysis, Writing – review & editing. JP: Formal Analysis, Writing – review & editing. TS: Formal Analysis, Writing – review & editing.
